# Analytical sociology and computational social science

**DOI:** 10.1007/s42001-017-0006-5

**Published:** 2017-11-21

**Authors:** Marc Keuschnigg, Niclas Lovsjö, Peter Hedström

**Affiliations:** The Institute for Analytical Sociology, Linköping University, Norra Grytsgatan 10, 601 74 Norrköping, Sweden

**Keywords:** Computational methods, Explanation, Induction and deduction, Online experiments, Social dynamics, Social mechanisms

## Abstract

Analytical sociology focuses on social interactions among individuals and the hard-to-predict aggregate outcomes they bring about. It seeks to identify generalizable mechanisms giving rise to emergent properties of social systems which, in turn, feed back on individual decision-making. This research program benefits from computational tools such as agent-based simulations, machine learning, and large-scale web experiments, and has considerable overlap with the nascent field of computational social science. By providing relevant analytical tools to rigorously address sociology’s core questions, computational social science has the potential to advance sociology in a similar way that the introduction of econometrics advanced economics during the last half century. Computational social scientists from computer science and physics often see as their main task to establish empirical regularities which they view as “social laws.” From the perspective of the social sciences, references to social laws appear unfounded and misplaced, however, and in this article we outline how analytical sociology, with its theory-grounded approach to computational social science, can help to move the field forward from mere descriptions and predictions to the explanation of social phenomena.

## Introduction

During the last decade and a half, analytical sociology (AS) has emerged as an increasingly important subfield of sociology. Substantively, it centers around the explanation of collective dynamics, and methodologically it is founded on the position that in order to explain collective dynamics, one must study the collectivity as a whole but not as a collectivity because patterns at the collective level rarely say much about the mechanisms that brought them about. Only by considering the individuals that are part of the collectivity, the relations between them, and their activities, can we explain the collective outcomes we observe [1].

More recently, the field of computational social science (CSS) has emerged that “leverages the capacity to collect and analyze data with an unprecedented breadth and depth and scale” [2]. CSS is located at the “intersection of the social and computational sciences, an intersection that includes analysis of web-scale observational data, virtual lab-style experiments, and computational modeling” [3]. Similar to AS, CSS perceives social phenomena as a result of social interactions among individual agents who are embedded in complex social systems and this poses considerable explanatory challenges because “the behavior of entities at one ‘scale’ of reality is not easily traced to the properties of the entities at the scale below” [3].

AS and CSS have much in common and there are important synergies to be gained from establishing closer ties between them. AS greatly benefits from the computational tools that are emerging from the CSS community such as agent-based simulations, machine learning, and large-scale web experiments. But AS and CSS differ considerably from one another in terms of their explanatory orientations and ambitions. After summarizing the core characteristics of AS and the numerous ways it benefits from the kind of computational tools associated with CSS, we outline how AS can contribute to the development of a more theory-grounded CSS that moves beyond predictions and descriptions of social regularities and seeks to identify the types of mechanisms responsible for their emergence.

## Defining characteristics of analytical sociology

AS emerged in part as a reaction to what was seen as an overly empiricist and descriptive stance among quantitatively oriented social scientists [4]. Descriptions of distributions and associations between abstract social categories or variables to a large extent had come to replace a focus on concrete actors, their relations, and the ways in which they bring about various aggregate outcomes. Since its beginnings in the early 2000s, AS has become increasingly diversified in terms of the topics being studied and the type of data and methods being used. The core defining characteristics of AS have changed very little, however, and can be summarized under three headings related to the importance of explanation, the modeling of dynamic social processes, and realism (for more detailed accounts of AS see [1, 4–6]).

### Explanation

Explanation in quantitative social science has become closely tied to the idea of establishing causal effects, and an appropriate explanation is often seen as a statement referring to prior events (or variables) that affect the probability of the event to be explained [7, 8]. The AS agenda is much more ambitious in terms of the type of explanations it seeks to develop. According to AS it is not sufficient to refer to causal associations between events. Rather, the mechanisms through which one event (or set of events) bring about the outcome to be explained must also be provided [9]. There exists an extensive philosophical and social-science literature on mechanisms and mechanism-based explanations [5], but Glennan and Illari’s generic and minimal definition captures the core idea. According to them a “mechanism for a phenomenon consists of entities (or parts) whose activities and interactions are organized as to be responsible for the phenomena” [10], and we explain a phenomenon by referring to the mechanism responsible for its production. In AS, the phenomena to be explained typically are important aggregate or macro outcomes such as network structures, segregation patterns, inequalities, cultural tastes, and common ways of acting. The entities we refer to in the explanation typically are individuals, and the activities referred to are the behavior of these individuals.

### Modeling social processes

Making sense of the relationship between micro behavior and macro outcomes thus is one of the central concerns of AS because the outcomes to be explained refer to properties of collectivities (or aggregates) such as groups, organizations, markets, and cities, while the explanatory mechanisms refer to individuals, their behavior, and the way in which the interaction between them is organized. The processes are complex because of mutual feedback—the macro properties of the system are the result of the behavior of the individuals, and the behavior of the individuals are in part the result of the properties of the system. AS, further, uses network metaphors, models, and measurements to characterize the ways in which interactions are organized. Although it is possible to establish regularities at the aggregate system or macro level, descriptions of such regularities often say very little about how the regularities were brought about. One striking example is Schelling’s segregation model which shows that extreme segregation can be brought about also by persons who individually are not segregationist in their preferences [11]. For such reasons, the guiding idea of AS is that we explain collective or aggregate outcomes: (1) by developing a model—possibly a simulation model—that shows how individuals can bring about such an outcome, and (2) by empirically demonstrating that the proposed model indeed is plausible in the specific case.

### Realism

Since social phenomena tend to be highly complex, some sort of generative model thus typically is required to understand how that which is to be explained was brought about, but if the model does not properly map onto reality, it is of little explanatory use. In contrast to empiricist and instrumentalist views, AS regards explanation as the principal epistemic aim of science, and AS regards explanation as factive. It is not enough that the theory or model predicts the phenomena to be explained; an explanation must represent the essential features of the actual process that produced the phenomena to be explained. Thus, AS does not accept the as-if attitude traditionally displayed by many economists [12], and from the AS perspective, considerations of elegance, simplicity, or tractability never should override the aim of accurately describing the mechanisms that actually produced the phenomena to be explained.

One study that nicely illustrates the explanatory approach of AS is Bearman and colleagues’ [13] investigation of sexual and romantic networks of adolescents. The context of their study was a high school in the United States, and the macro structure they sought to explain was the surprising discovery that students’ sexual and romantic network resembled a spanning tree. They identified different micro-level mechanisms that potentially could explain this macro-level pattern and used simulations to derive what the network structure would have looked like if a particular micro mechanism had been at work. By performing different simulations, they came to the conclusion that the spanning-tree structure most likely was the result of a social norm that prohibited dating cycles of a length of four. From a boy’s point of view, this norm implied that he should not form a partnership with his prior girlfriend’s current boyfriend’s prior girlfriend, and vice versa for the girls. Unlike the other mechanisms they considered, postulating the existence of such a norm would bring about the observed network structure, and the norm seemed empirically plausible in the social system that they studied. By identifying the macro-outcome and the dynamic process by which it emerged, Bearman and colleagues were able to shed new light on network formation and how to design interventions to prevent the spread of sexually transmitted diseases.

## Computational tools as the econometrics of sociology

Until very recently sociologists did not have the analytical tools needed for analyzing the dynamics of complex systems that large groups of interacting individuals represent. Survey data and the statistical tools developed for analyzing such data instead came to dominate empirical social analysis. As noted by Coleman [7], one of the most important intellectual forefathers of AS, it is puzzling that a technique developed for analyzing the behavior of samples of independent individuals came to dominate a discipline primarily concerned with social systems comprised of interacting and interdependent individuals. According to Coleman, this tension led a to a widening gap between theoretical and empirical work because empirical researchers gradually started to focus on research questions that the new tools could address. As a consequence, there was a shift in focus from the “social processes (…) shaping the system’s behavior to psychological and demographic processes shaping individual behavior” [7].

The times are changing, however, and tools for analyses of systems consisting of large numbers of interacting individuals are being developed. Powerful computers in combination with the digitalization of the social world allow for rigorous empirical analyses of large and complex social systems. These new tools and data sources make it possible to address the traditional core questions of the discipline in an equally rigorous fashion as survey-based researchers were able to answer questions about the behavior of independent individuals.

Empirically calibrated agent-based models are one important tool that exemplify this development [14–16]. In order to avoid basing the analysis on implausible or arbitrary assumptions that could threaten the explanatory power of the analysis, detailed empirical analyses are used to inform the specification of the agent-based simulation model and to decide upon realistic parameter values. Using such models, it becomes possible to assess whether the system outcome to be explained could have been produced in the postulated manner and to assess how interventions on micro parameters are likely to change the system’s macro dynamics. The empirically calibrated simulation model can then be used as a “societal flight simulator” in contexts defying actual manipulation due to costs, practical limitations, or ethical concern [17, 18].

Large-scale experiments implemented over the Internet are additional CSS tools that are of considerable importance for AS because they allow for truly macro-sociological experiments [19, 20]. To shift the experimental focus from individual behavior to system behavior requires designs that break from tradition. In these macro-sociological experiments, large and heterogeneous groups of individuals represent a set of separate social systems that serve as the units of analysis, and data collection captures social processes evolving over time. The counterfactual approach [21, 22] then leverages the comparison of multiple realizations of a collective process in both large-scale virtual labs and field implementations [23–25].

Rich observational data from mobile apps, social networking sites, discussion forums, and commercial platforms also represent an important development in that they constitute highly granular, time-stamped information for sociological inquiry [26]. This type of data identifies not only the choices of individuals but also how individuals consider alternatives, learn, adjust, and ultimately decide against certain options [27]. From the standpoint of causal inference, these datasets permit longitudinal analyses of infinitesimal period length and high-dimensional matching relying not only on a handful of sociodemographic variables but hundreds or thousands of individual and contextual attributes. This type of data is inherently different from the data traditionally used in the social sciences. Among social scientists, this has spurred interest in new methods including the vibrant field of machine learning [28]. By relaxing statistical assumptions researchers in this field are now able to analyze datasets of extreme size and depth. But this comes at a cost. Instead of focusing on parameter estimation of models built from theory, machine learning models are typically evaluated on their ability to predict held-out samples of the data. Unlike the predictive use of machine learning common among computer scientists, social scientists start employing machine learning to measure latent characteristics in the social world and refine methods of causal inference from observational data [29–32].

Much social data comes in textual form. During the last 20 years there has been a surge of methods developed for processing text under the label of natural language processing [33] including tools for the classification of content in large documents [34], semantic analysis [35], and opinion mining [36]. These tools, now readily available to researchers in the social sciences, make text originating from a wide range of sources such as books, newspapers, and online discussion groups accessible for large-scale quantitative analysis [29]. As becomes evident from a growing number of applications in sociology and the social sciences more generally, these powerful tools open new avenues for an ethnography on a systematic scale including, to name but a few, the study of culture [37, 38], analyses of political expression and conflict [39, 40] and, in combination with digitized archives, investigations of historical events and social change [41, 42].

We believe that explorative methods of pattern recognition in unstructured texts and digital traces have the potential to overcome the qualitative—quantitative divide in the social sciences and vindicate inferential approaches such as grounded theory [43], abductive analysis [44], and forensic social science [45] among quantitatively-orientated sociologists [46, 47]. Most quantitative social scientists are skeptical about data mining which—clearly at odds with the Popperian recipe for scientific progress [48]—is suspected for “fishing” for effects and for tailoring of post hoc explanations fitting the specific dataset at hand. Given the current state of social theory, however, it would be a grave mistake to ignore these tools for data-driven explorative analyses when seeking to gain a better understanding of social dynamics.

In our view, CSS has the potential to accomplish for sociology what the introduction of econometrics did for economics in the past half century, i.e., to provide the relevant analytical tools and data needed to rigorously address the core questions of the discipline. Unlike the case of economics—where the analysis of individual outcomes remains pivotal—the main concern of sociology in general and AS in particular are explanations of macro patterns and dynamics that emerge from individuals’ interactions. For such analyses, traditional econometrics with its focus on either micro-level or macro-level analyses does not readily apply. Moreover, simple extrapolation from individual-level analyses is impossible because the nature and consequences of social interactions change as one moves from one scale to another [49]. The new CSS-related data sources and analytical tools provide an excellent fit with a sociological tradition interested primarily in the explanation of networked social systems and their dynamics.

## What does analytical sociology add to computational social science?

The close relation between CSS and AS is not a one-way street, and in this section, we discuss what we see as the most important contributions of AS to the CSS agenda. Reviewing the programmatic publications in the field, we cannot escape the conclusion that mainstream CSS follows a descriptive and predictive approach to social phenomena, with little or no attention paid to the mechanisms through which social outcomes are brought about. Lazer et al.’s highly-cited memorandum [2], for example, is strongly descriptively orientated and Cioffi-Revilla’s overview [50] is exclusively concerned with the descriptive and predictive uses of CSS methods.

The descriptive impetus of CSS typically centers around identifying universal patterns which, at times, are characterized as “social laws.” The detection of power-law distributions in different domains of social life [51–53] is an important example of this. The finding of power laws is commonly interpreted as an indication of out-of-equilibrium systems [54] and, far too often, researchers claim that, whenever you find a power law, similar mechanisms are at work [52, 55, 56]. A similar focus and a similar type of inference are common in network science [57, 58]. Focusing on aggregate patterns such as power laws, however, does not lead to the identification of “social laws” but rather results in descriptions of regularities that lack causal depth. Trying to make any sort of causal inference on the basis of macro-level regularities is highly error prone. Discriminating between different generative mechanisms based on aggregate data alone becomes impossible if those processes lead to very similar macro patterns [59]. Stumpf and Porter [60] similarly emphasized that “a statistically sound power law is no evidence of universality without a concrete underlying theory to support it”, and researchers should not imbue them “with a vague and mistakenly mystical sense of universality”. In our view, CSS would greatly benefit from a realistic and mechanism-based focus similar to that found within AS because it would lead to more nuanced research and better explanations of why we observe what we observe.

We have many fewer concerns when it comes to the applied uses of CSS. The real-time collection of online data is important because it allows for analyses of issues of high public relevance such as the early detection of outbreaks of contagious diseases based on web search queries and symptom posting on social media [61–63]. In an exemplary study on effective vaccination strategies, Mones et al. [64] equipped Danish students with mobile phones to collect multidimensional network data from proximate and online interactions. The authors ran virtual experiments randomly “infecting” some individuals and “vaccinating” others. Online network data that normally would be available in the case of a real epidemic, was then used to determine central nodes in the student network. In comparison to standard vaccination strategies, selective vaccination of individuals who were central in the online network considerably improved outbreak containment.

Groundbreaking and important applications such as these have led to some provocative claims about the end of theory: “The new availability of data, along with the statistical tools to crunch these numbers, offers a whole new way of understanding the world. Correlation supersedes causation, and science can advance even without coherent models, unified theories, or really any mechanistic explanation at all” [65]. From the viewpoint of AS, this radical form of empiricism is indefensible. Social scientists, of course, should not rest with finding correlations, but should aim for explanations that refer to the actual causal mechanisms at work.

A theory-driven approach is important because it helps us to ask the right questions and to make sense of empirical results [66]. Although it is true that large-scale and fine-grained data leave less room for researchers own interpretations of empirical findings, and that direct tests of the underlying causes of observed phenomena sometimes are possible [27], interpretations of causal effects and the transportability of results from one domain to another require that the type of generative mechanism is identified. If results cannot be traced back to general mechanisms, understanding remains incomplete and generalization beyond the specific case becomes hard to attain [5, 67, 68].

A focus on mechanism-based explanations is important not just because it produces intellectually more satisfying explanations, but also because it makes it easier to isolate effective policy interventions. Although many predictive models directly translate to powerful policy recommendations, it is important to note that interventions such as those described above that are concerned with the spreading of a contagious disease, take place in the physical world where we have remarkable understanding of how things work. Predictive models as such are black boxes which do not add anything to this understanding but feed on it. For interventions in the social world, e.g., in financial markets or political systems, we lack the corresponding knowledge that would allow us to directly turn predictions into effective interventions. We might be able to predict stock market shifts from Twitter moods [69] or foresee political instability from social media postings [70], but to intervene in these sorts of developments we need to understand how the systems work. Not until we better understand the functioning of social systems and how changes in some of its parameters may change relevant aggregate properties of the system, have we identified potential intervention points and policy levelers.

Leading figures in the CSS community call for a similar epistemological foundation as the one advocated here [3, 71, 72]. Conte et al. [72], for example, state that “(u)nlike predictive uses, which primarily involve optimizing the models to make their output as accurate and precise as we need it to be, the explanatory use requires us to learn how the component parts of the target system give rise to the behavior of the whole.” This vision of CSS is highly compatible with the AS research program.

An important barrier for effective communication and collaboration between researchers in AS and CSS centers on the issue of domain knowledge. Sociologists have been working on the description and explanation of social phenomena for a century now, and as Mützel emphasizes, “because of its insights and techniques to study meaning and how the social is constructed, sociology makes itself very relevant to data science projects mining large data sets” [73]. So far, however, mainstream CSS has paid minimal attention to sociology (and the other social sciences), and according to Watts’ [3] diagnosis, the relationship is reciprocal: “much of computational social science has effectively evolved in isolation from the rest of social science, largely ignoring much of what social scientists have to say about the same topics, and largely being ignored by them in return.”

## Conclusion

The tremendous growth of survey-based research has profoundly changed the character of sociological research. While sociological research traditionally focused on communities, neighborhoods, and other systems of interaction, the type of quantitative survey research that has come to dominate the discipline, largely focuses on the socio-demographic and psychological “determinants” of individual behavior. Coleman [7] expressed deep concerns about this development and described it as a “watershed” in empirical sociological research. The increasing availability of fine-grained data from various online sources combined with the rapid developments in computationally intensive statistical methods and data analysis are exciting from a sociological perspective; they suggest that we are on the verge of a second watershed [45] that will make empirical research better aligned with sociological theory and its focus on the hard-to-predict aggregate outcomes that interacting individuals bring about.

The growing availability of CSS-related tools also is likely to help eradicating the rather unproductive quantitative–qualitative divide within sociology. New explorative methods for finding patterns in unstructured texts and digital traces, for example, hold the promise of a more systematic and rigorous type of inductive research than what existing qualitative approaches allow for.

In this paper, we have argued that CSS has much to gain from abandoning its current search for “social laws” and instead adopting the realist and mechanism-oriented explanatory approach of AS. Identifying how various types of mechanisms bring about system-level outcomes contributes to the theoretical development of the social sciences and also is important for devising effective policy interventions.

Seeking to identify the types of mechanisms that explain various social phenomena is far from trivial, however, and can easily lead to mere “mechanism talk” [74] and common-sense storytelling [75]. Mechanisms need to be predictive in order to be taken seriously, but this does not imply that we embrace the use of prediction and as-if models. Instead, we believe that computational tools such as agent-based simulations, macro-oriented experiments, counterfactual approaches to causality, and inductive analyses using machine-learning techniques to be important parts of this endeavour [21, 75–77].

To make progress on identifying the mechanisms that operate in large-scale interactive systems requires skill sets from the social sciences as well as the computational sciences. As expressed by Watts [3]: “meaningful progress on important problems will require serious engagement between the communities, each of which has much to offer the other: computer scientists have technical capabilities that are of great potential benefit to social scientists, and the latter’s deep subject matter knowledge is essential in order to ask the right questions and to formulate even simple models in ways that address these questions.”

As noted above, we have not yet seen much of such interdisciplinary collaboration, however. In part, this may be due to that social systems are quite different from physical systems, and that both computer science and physics with their emphasis on predictive power offer a poor template for social inquiry [78]. But in our view, the lack of interdisciplinary collaborations rather is due to the newness of the field. Breaking down disciplinary boundaries between the social sciences and the computational sciences will take time, but the emergence of both interdisciplinary CSS conferences and journals combined with the obvious payoffs that the establishment of such collaborations offer make us very hopeful for the future.
